# Determination of Lipid Hydroperoxides in Marine Diatoms by the FOX2 Assay

**DOI:** 10.3390/md13095767

**Published:** 2015-09-11

**Authors:** Ida Orefice, Andrea Gerecht, Giuliana d’Ippolito, Angelo Fontana, Adrianna Ianora, Giovanna Romano

**Affiliations:** 1 Chemical Ecology Laboratory, Integrative Marine Ecology Department, Stazione Zoologica Anton Dohrn, Villa Comunale, 80121 Naples, Italy; E-Mails: adrianna.ianora@szn.it (A.I.); giovanna.romano@szn.it (G.R.); 2 Faculty of Biosciences, Fisheries and Economics, UiT-The Arctic University of Norway, P.O. Box 6050 Langnes, 9037 Tromsø, Norway; E-Mail: andrea.gerecht@uit.no; 3 Bio-Organic Chemistry Unit, Institute Biomolecular Chemistry-CNR, Via Campi Flegrei 34, Pozzuoli, 80078 Naples, Italy; E-Mails: giuliana.dippolito@icb.cnr.it (G.D.); angelo.fontana@icb.cnr.it (A.F.)

**Keywords:** diatoms, diatom detrimental effect, fatty acid hydroperoxides, FOX2 assay, lipoxygenase, oxylipins

## Abstract

Ecologically-relevant marine diatoms produce a plethora of bioactive oxylipins deriving from fatty acid oxidation, including aldehydes, hydroxy-fatty acids, epoxy-hydroxy-fatty acids, and oxo-acids. These secondary metabolites have been related to the negative effect of diatoms on copepod reproduction, causing low hatching success and teratogenesis in the offspring during periods of intense diatom blooms. The common intermediates in the formation of oxylipins are fatty acid hydroperoxides. The quantitative measurement of these intermediates can fundamentally contribute to understanding the function and role of lipoxygenase metabolites in diatom-copepod interactions. Here, we describe the successful adaptation of the ferrous oxidation-xylenol orange 2 (FOX2) assay to diatom samples, which showed several advantages over other spectrophotometric and polarographic methods tested in the present work. Using this method we assessed fatty acid hydroperoxide levels in three diatom species: *Skeletonema marinoi*, *Thalassiosira rotula*, and *Chaetoceros affinis*, and discuss results in light of the literature data on their detrimental effects on copepod reproduction.

## 1. Introduction

Diatoms have traditionally been regarded as a beneficial food source for calanoid copepods [1]. Only rather recently has this paradigm been challenged, as more and more evidence is uncovered as to the negative effects that this class of microalgae can have on the reproductive success of their main grazers [2,3]. When copepods feed on certain diatom species, their eggs either fail to hatch or hatch into malformed nauplii [4,5]. Low hatching success of copepod eggs during periods of intense diatom blooms has also been observed in the field [6,7,8]. Deleterious effects of diatom diets have been ascribed to the oxidation of polyunsaturated C_16_- and C_20_-fatty acids (PUFAs) to short-chain polyunsaturated aldehydes (PUAs) [6] and other non-volatile oxylipins (NVOs), such as hydroxy-fatty acids, epoxy-hydroxy-fatty acids, and oxo-acids [9,10]. However, the role that these oxidized fatty acid derivatives play in diatom-copepod interactions is still under discussion. Production of oxylipins is triggered by cell damage, as occurs during grazing or lysis of the cells [11]. Fatty acids, liberated from cell membranes, are first oxidized by specific lipoxygenases (LOXs) to fatty acid hydroperoxides (FAHs), which are rapidly converted to PUAs and NVOs [12]. Although the LOX protein has not yet been isolated in diatoms, oxidation of polyunsaturated fatty acids occurs with a high regio- and stereo-specificity, indicating a tight enzymatic control of the process, analogous to terrestrial plant LOX pathways [12]. As in plants and animals, hydroperoxides produced in diatoms can follow different pathways that give rise to a plethora of different end-products depending on the downstream enzymes.

Recent studies have highlighted differences in the oxylipin metabolism at the species and even clone level in diatoms [12,13], resulting in the production of several distinct end-metabolites. In certain diatom species, the chemical profiles of oxylipins corroborate the genotypic delineation, even among genetically closely-related cryptic species [14]. This suggests that oxylipin profiles may constitute an additional taxonomic identification tool, providing a functional support to species delineation obtained by molecular markers and morphological traits [14]. Differences among diatom species in the potency of interfering with reproductive processes in copepods have been reported in previous papers [3,4,10]. This diversity may be related to the variations in oxylipin metabolism and in oxidative processes triggered in the diatom cells by external signals or cell damage. The fundamental step in the above scenario is the oxidation of PUFAs by LOXs, with the production of FAHs as a first product. FAHs are prominent non-radical intermediates of lipid peroxidation. Being more polar than parent lipids, they perturb membrane structure/function and can be deleterious to cells on this basis alone, or can participate in redox reactions. Lipid peroxidation may evoke a variety of cellular responses, ranging from induction of antioxidant enzymes to apoptotic death [15]. FAHs produced by diatoms upon cell breakage have been shown to be deleterious to copepod development at concentrations comparable to those of polyunsaturated aldehydes [10]. The activation of the LOX pathways is the first event of the oxidative burst occurring in a diatom following cell rupture as demonstrated by the stereochemical purity of diatom-produced hydroperoxyeicosapentaenoic acids and hydroxyeicosapentaenoic acids, thus excluding the possibility that lipid peroxidation could be initiated by free radicals [10]. The pool of molecules generated by the LOX-initiated process upon cell rupture is responsible for the damage in copepod females that feed on these diatoms. The extent of oxidative damage may overwhelm the repair capacity in the copepod and induce apoptosis or necrosis in many tissues, especially in gonads [16,17] resulting in reproductive failure.

The evaluation of the key FAH intermediates in cultured diatoms and in natural field samples, may give important insights into the role of the lipid oxidative pathway in diatom-copepod interactions. The most commonly used method to measure FAH production is the spectrophotometric determination of absorbance at 234 nm due to the formation of conjugated dienes present in oxidised PUFAs [18]. However, this method is not feasible for diatom crude cell lysate because many other substances strongly absorb in the UV range. A polarographic assay, which is based on oxygen consumption during PUFA oxidation, has also been used [19]. This assay is very accurate but needs specialized equipment, such as an oxygen electrode, and requires strict control of oxygen levels in the reaction mixture. Alternatively, one can measure specific oxylipins as end-products of LOX pathways by chromatographic and spectrometric techniques [20]. This approach has been the most frequently used to assess putative diatom toxicity. Although the chemical analysis of oxylipins (PUAs, NVOs) is a precise and sensitive method, it is neither rapid nor cost effective. Moreover, the degradation of hydroperoxides produces many different molecules. Thus measuring only the known end-products can lead to an underestimation of lipid peroxidation.

**Figure 1:**
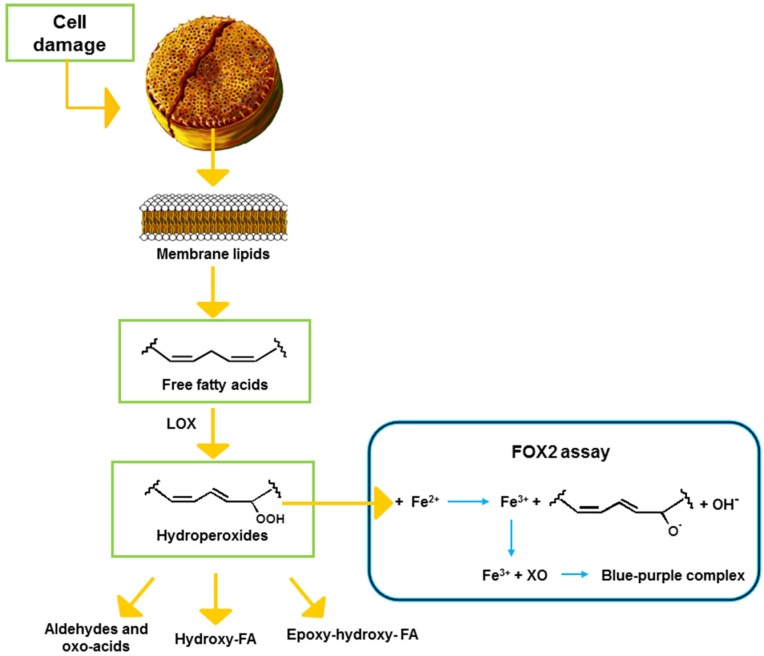
Schematic representation of the lipoxygenase (LOX) pathway in diatoms and the FOX2 reaction. FA: fatty acid, XO: xylenol orange.

Colorimetric assays can also be used to measure FAHs in samples of various origins. Among these, the method developed by Anthon and Barrett [21] has been previously used to determine LOX activity by FAH production in plants, and more recently also in diatoms [9,10,22]. The aim of the present study was to evaluate an alternative colorimetric assay, requiring standard laboratory equipment, to predict potential toxicity of diatoms and to compare it to other commonly used assays. To this end, we adapted the ferrous oxidation-xylenol orange 2 (FOX2) assay that is based on the oxidation of reagent iron (II) to iron (III) by oxidizing agents present in the sample (Figure 1). The reagent iron (III) then binds to the xylenol orange reagent giving a color complex with an absorbance maximum at 560 nm.

Compared to the FOX1 assay, which is suitable for the determination of low levels of hydrogen peroxide in aqueous buffers, the FOX2 assay is more specific for the determination of FAHs [23] and allows the quantification of low concentrations of these molecules in the presence of high background levels of non-peroxidized fatty acids [24]. Moreover, in the FOX2 assay the use of the antioxidant butylated hydroxytoluene (BHT) prevents the generation of further hydroperoxides by a chain reaction [23]. This assay is fast, sensitive, precise, and inexpensive and it has a broad applicability [25]. It has been successfully applied to diverse biological samples [26,27], including fats and oils [28,29,30], lipid extracts, homogenates from meat and vegetables [24,31,32], and for detecting the incipient lipid peroxidation in macroalgal tissues [33]. Furthermore, in combination with HPLC fractionation, the method has been recently used for determination and quantification of different classes of lipid hydroperoxides in organic matrices [34]. Here we evaluated application of FOX2 to measure FAHs in three diatom species, the well-studied PUA-producing species *Skeletonema marinoi*, *Thalassiosira rotula*, and *Chaetoceros affinis*, which produces only NVOs [10,12,35]. We interpret our results in the light of data on the detrimental effect of these diatoms on copepod reproduction previously obtained in our laboratory and available in the literature (see review by Ianora and Miralto [3]).

## 2. Results

### 2.1. Optimization of the FOX2 Assay for Diatom Samples

Using *S. marinoi* as a model species, the FOX2 assay was adapted to diatom samples. The kinetics of FAH production was very fast, reaching a plateau after 5 min from sonication (Figure S1) that remained stable up to 20 min. On the basis of these results, the reading of absorbance was standardized to 10 min after sonication. Shorter incubation times may increase the influence of differences in sampling time, possibly generating less reproducible results.

**Figure 2:**
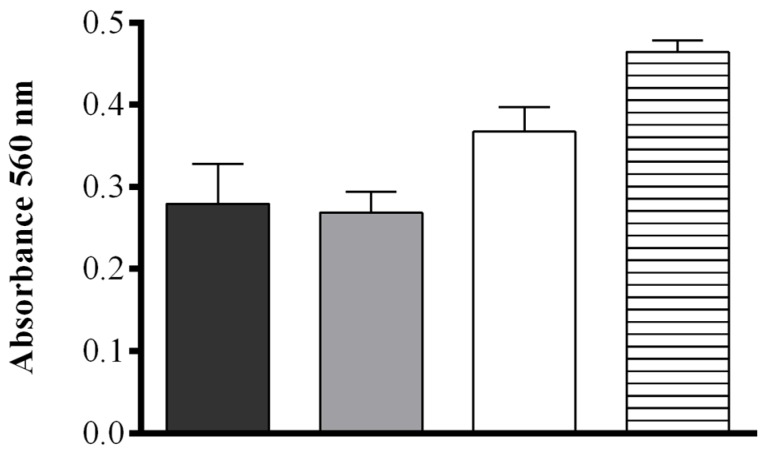
Effect of four different extraction media. Deionized water (**black**), 50 mM Tris-HCl pH = 7.50 (**gray**), 50 mM Tris-HCl pH = 8.15 (**white**), and 50 mM Tris-HCl pH = 8.15 containing 0.5 M NaCl (**hatched**), on hydroperoxide production in *Skeletonema marinoi* lysate at 10 min after sonication using the FOX2 assay (shown as absorbance at 560 nm). Data represent mean + SD of three biological replicates.

The effect of pH and salt concentration were also assessed. No significant difference in the production of FAHs was detected between samples sonicated in deionized H_2_O (MilliQ) or in 50 mM Tris-HCl at pH = 7.50 (0.279 ± 0.048 and 0.268 ± 0.026 OD, respectively), while at increasing buffer pH (8.15) a more intense absorbance was observed (0.367 ± 0.029 OD). When pellets of *S. marinoi* were suspended in 50 mM Tris-HCl at pH = 8.15 containing 0.5 M NaCl, the production of FAHs was significantly higher upon sonication than for all other conditions tested (0.464 ± 0.013 OD) (one-way ANOVA: *F* = 24.74, *r^2^* = 0.9027, *p* = 0.0002) (Figure 2). The contribution of other components of the *S. marinoi* lysate to the absorbance at the wavelength used in the assay (560 nm) was insignificant for samples suspended in this buffer and therefore did not interfere with the FOX2 assay (Figure S2).

**Figure 3:**
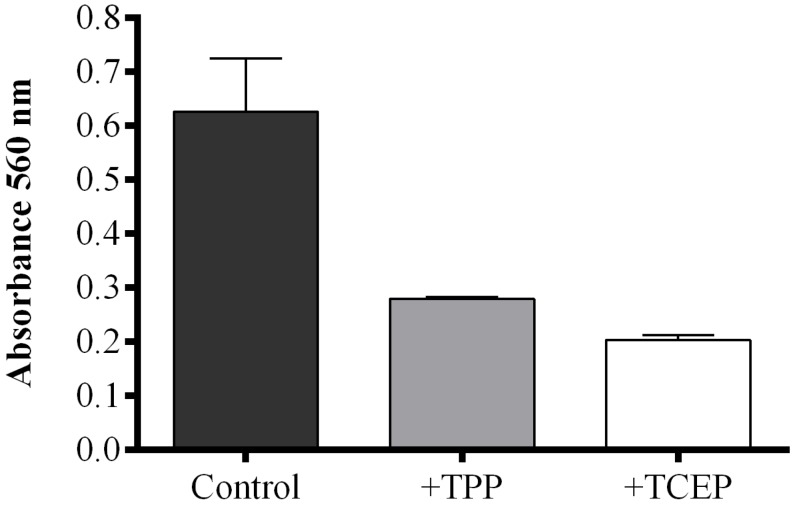
Effect of reducing reagents on fatty acid hydroperoxide production. The FOX2 assay was performed on *Skeletonema marinoi* lysate. Aliquots were incubated with either 40 μL methanol (control incubation, black), with 2 mM triphenylphosphine (TPP, grey), or with 50 mM tris(2-carboxyethyl)phosphine (TCEP, white). Data are reported as absorbance at 560 nm and represent mean + SD of three biological replicates.

When the reducing agent triphenylphosphine (TPP) was added to *S. marinoi* samples, FAHs were reduced to the corresponding hydroxyacids with a consequent decrease of absorbance compared to TPP-untreated samples. However, the reaction mixture became opalescent, interfering with the correct reading of absorbance. This problem was solved by using a reducing reagent soluble in water, tris(2-carboxyethyl)phosphine (TCEP). To compare the reducing activity of TPP and TCEP, the FOX2 assay was also performed on commercial LOX incubated with linoleic acid (LA) as substrate. The FOX2-generated absorbance was completely abolished by pre-incubation with 2 mM methanolic TPP solution, while the same result was obtained with 50 mM TCEP solution after one hour of incubation (Figure S3). Both reducing reagents efficiently converted FAHs to hydroxyacids in *S. marinoi* lysate, with a consequent reduction of absorbance compared to untreated samples (Figure 3). The reducing reagent TCEP seemed to be more efficient in reducing FAHs generated in diatom lysates inducing a more pronounced decrease in absorbance than TPP-treated samples.

### 2.2. Fatty Acid Hydroperoxide Production in Selected Diatom Species

**Figure 4:**
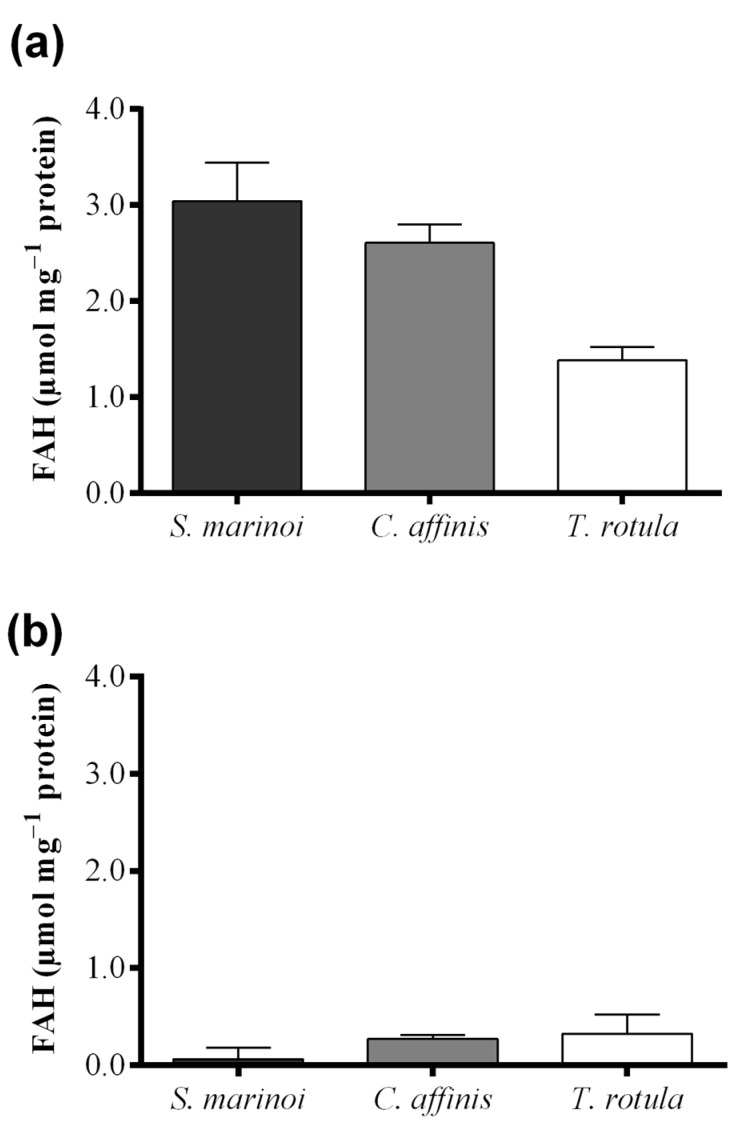

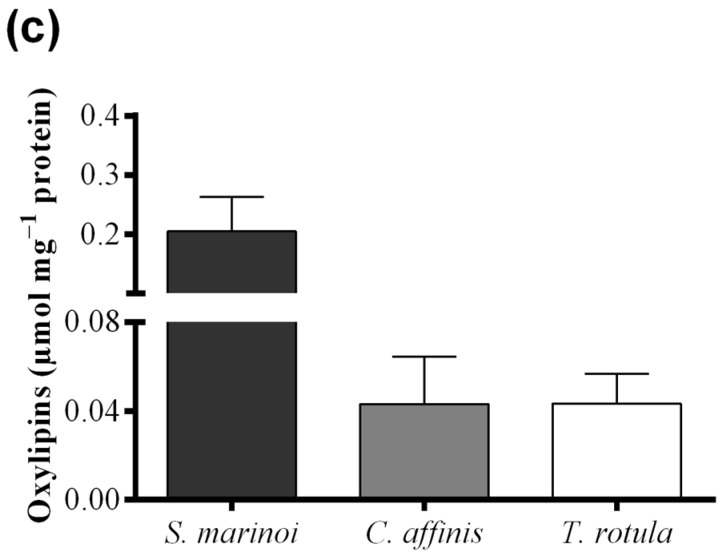
Comparison among diatom species, *Skeletonema marinoi* (black), *Chaetoceros affinis* (grey) and *Thalassiosira rotula* (white), using three different methods. (**a**) Hydroperoxide production expressed as μmol fatty acid hydroperoxides per mg protein measured with the colorimetric FOX2 assay. Data were obtained as the difference between tris(2-carboxyethyl)phosphine treated and untreated samples; (**b**) Hydroperoxide production expressed as μmol fatty acid hydroperoxides per mg protein measured at pH = 6.00 with the colorimetric “Anthon and Barrett” assay and (**c**) oxylipin production expressed as μmol per mg protein quantified by chromatography mass spectrometry (LC and GC–MS). Data represent the mean + SD of at least three biological replicates.

*Skeletonema marinoi* and *C. affinis* showed the highest levels of FAHs (3.04 ± 0.69 and 2.61 ± 0.33 μmol·mg^−1^ protein, respectively) with no significant difference between the two species (Figure 4a and Table 1). *Thalassiosira rotula* exhibited about 50% lower production of FAHs, at 1.39 ± 0.23 μmol·mg^−1^ protein. For *S. marinoi* and *C. affinis* we estimated a range of applicability of the FOX2 assay between 2 and 20 μg of protein, whilst for *T. rotula* the range was shifted towards higher protein content, between 4 and 50 μg.

Hydroperoxide levels measured with the colorimetric assay of “Anthon and Barrett” were, instead, similar in *C. affinis* and *T. rotula* (0.28 ± 0.04 and 0.33 ± 0.20 µmol·mg^−1^ protein, respectively) (Figure 4b). *Skeletonema marinoi*, on the other hand, showed very low activity (0.07 ± 0.12 µmol·mg^−1^ protein). The variability among replicates was high in *T. rotula* and *S. marinoi* and more constrained in *C. affinis*. Although a significant LOX activity could not be demonstrated by means of this assay in *S. marinoi*, this species produced the highest levels of oxylipins (0.20 ± 0.06 µmol mg^−1^ protein) (Figure 4c and Table 1), while *C. affinis* and *T. rotula* produced lower, similar amounts of oxylipins (both circa 0.043 µmol mg^−1^ protein).

**Table 1:** Statistical results for the comparison of diatom sample pairs using Tukey’s Multiple Comparison Test. The table reports: the difference between samples (Mean Diff.), the *q* ratio and *p* value. Values for *p* < 0.05 are considered significant difference.

Sample Pairs	Mean Diff.	*q*	*p* Value
FOX2 assay			
*S. marinoi–C. affinis*	0.43	1.61	*p* > 0.05
*S. marinoi–T. rotula*	1.65	6.16	*p* < 0.05
*C. affinis–T. rotula*	1.22	4.55	*p* < 0.05
“Anthon and Barrett” assay			
*S. marinoi–C. affinis*	−0.21	3.44	*p* > 0.05
*S. marinoi–T. rotula*	−0.26	4.27	*p* < 0.05
*C. affinis–T. rotula*	−0.05	0.75	*p* > 0.05
LC and GC–MS			
*S. marinoi–C. affinis*	0.16	7.10	*p* < 0.001
*S. marinoi–T. rotula*	0.16	7.09	*p* < 0.001
*C. affinis–T. rotula*	−0.00023	0.0087	*p* > 0.05

**Figure 5:**
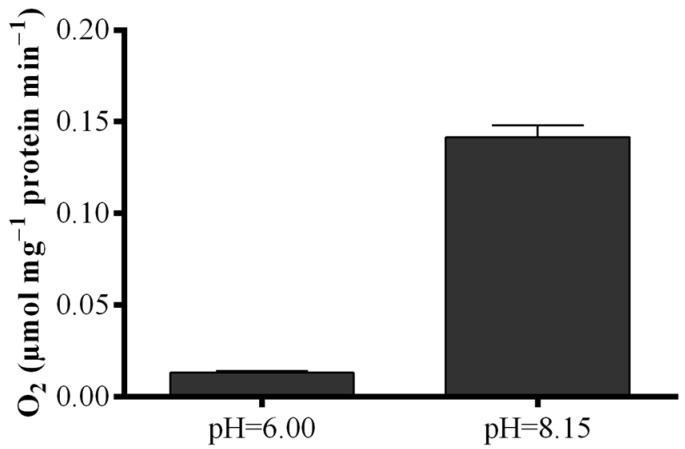
Dependence of fatty acid hydroperoxide production on pH conditions. Oxygen consumption at pH = 6.00 and pH = 8.15 upon addition of eicosapentaenoic acid to *Skeletonema marinoi* lysate expressed as µmol·mg^−1^ protein min^−1^. Data represent the mean + SD.

To elucidate the contradictory results obtained with the *S. marinoi* lysate, LOX activity was measured by means of a polarographic method with eicosapentaenoic acid (EPA) as substrate (Figure 5). Results revealed that oxygen consumption of the lysate at the pH of the “Anthon and Barrett” assay (pH = 6.00) was very low, while at pH = 8.15, oxygen consumption was one order of magnitude higher increasing from 0.013 ± 0.01 to 0.141 ± 0.06 µmol·mg^−1^ protein min^−1^.

### 2.3. Effect of Freezing on Diatom Samples

**Figure 6:**
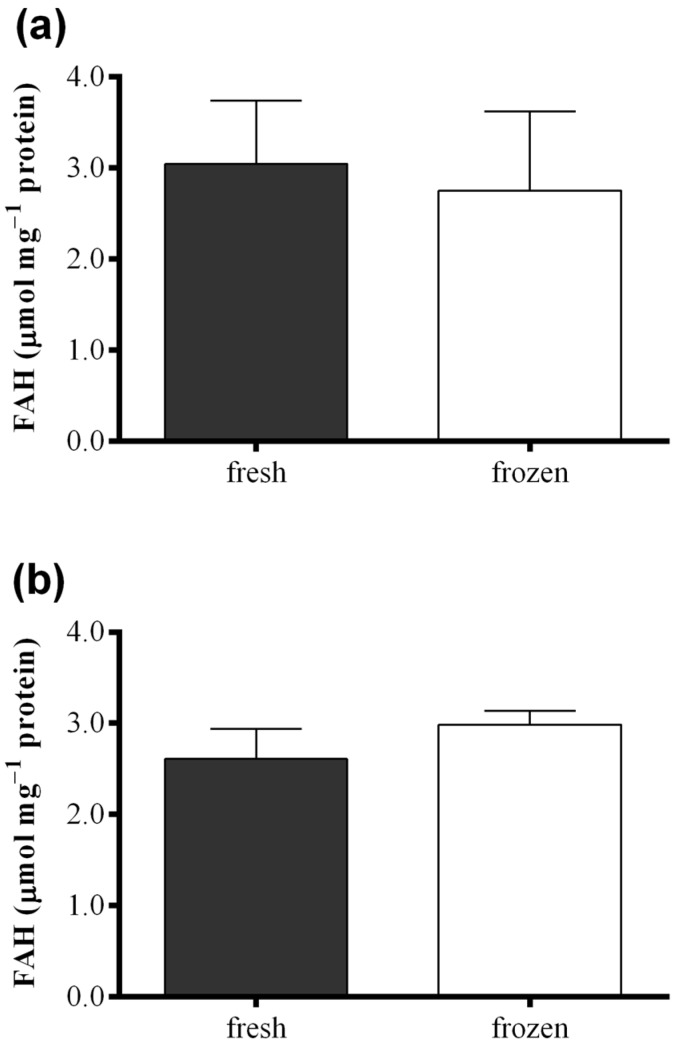

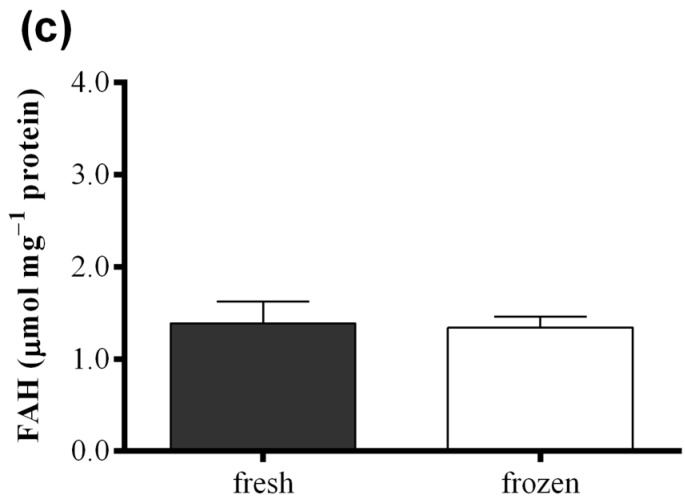
Comparison between fresh (black) and frozen (white) diatom samples. Fatty acid hydroperoxide production was measured with the FOX2 assay in samples of (**a**) *Skeletonema marinoi*; (**b**) *Chaetoceros affinis*; and (**c**) *Thalassiosira rotula*. Data were obtained as the difference between tris(2-carboxyethyl)phosphine treated and untreated samples and represent mean +SD of three biological replicates.

Figure 6 shows results obtained with the FOX2 assay performed on fresh samples of *S. marinoi*, *C. affinis*, and *T. rotula* and on frozen samples obtained from the same culture, but analyzed after storing one month at −80 °C. Differences in FAH concentration were insignificant between samples for all the three species analyzed (paired *t*-test: *p* > 0.05). The same comparison was also performed for the “Anthon and Barrett” colorimetric assay, the polarographic method, and oxylipin quantification for *C. affinis*, showing comparable values for fresh and frozen samples (Figure S4).

## 3. Discussion

In this study the FOX2 colorimetric assay was adapted to assess the levels of FAHs in diatom samples. Standard assays for measuring LOX activity in plants require the addition of exogenous PUFAs. However, diatoms contain large quantities of endogenous PUFAs [36] and it is, therefore, possible to apply the FOX2 assay to diatom samples without external substrate addition. This is a great advantage because certain diatom species, such as *S. marinoi* and *T. rotula*, channel LOX activity into two different pathways that require either C_16_- or C_20_-PUFAs as main substrates. Of these PUFAs, only EPA is commercially available. Using only this PUFA as exogenous substrate, the contribution of the C_16_-PUFA metabolism is overlooked and only a partial assessment of LOX activity is possible. This is also a limitation of the polarographic method that requires the addition of external substrate to differentiate oxygen consumption due to LOX activity from unspecific oxygen consumption in the lysate.

We obtained the best results with the FOX2 assay using conditions very close to that of seawater in terms of pH (8.15) and ionic strength, suggesting that the activity optimum of diatom LOX enzymes may lie in their surrounding environment. Using a reducing reagent compatible with saltwater, it was possible to subtract the background absorbance of potentially inferring substances [37,38]. Endogenous iron (III) may, in fact, conjugate with xylenol orange, leading to an overestimation of FAH levels. Another possibly interfering substance is hydrogen peroxide. A sample blank in which the FAHs in the sample are reduced is, thus, required to improve assay specificity. This was accomplished by treating the samples with a reducing agent to transform FAHs to the corresponding hydroxyacids. The hydrophobic TPP and the more polar TCEP specifically reduce lipid hydroperoxides but do not react with hydrogen peroxide [25,38], thus allowing to discriminate between hydrogen peroxide and other hydroperoxides in the samples. In this way the FOX2 assay can be used to specifically determine FAH concentrations in diatom extracts by measuring the difference in absorbance in the presence and absence of a reducing reagent.

Using the improved FOX2 assay, we could show that there are marked differences in LOX metabolism among the diatom species *S. marinoi*, *T. rotula*, and *C. affinis* with *S. marinoi* and *C. affinis* producing higher FAH concentrations than *T. rotula*. The colorimetric assay proposed by “Anthon and Barrett” gave different results for the same species with similar FAH concentrations in *C. affinis* and *T. rotula*. *Skeletonema marinoi* exhibited almost no production. Moreover, the “Anthon and Barrett” assay greatly underestimated FAH production in all species with values one order of magnitude lower than those obtained with the FOX2 assay. The replicability of results was also much lower. The lack of activity in *S. marinoi* in the “Anthon and Barrett” assay was surprising as this species produces high levels of oxylipins and has a strong negative effect on copepod hatching success [4,10]. By means of the polarographic assay and by varying buffer pH, we were able to show that the lack of FAH production was due to the assay conditions. While LOXs present in *S. marinoi* lack activity at the pH of the “Anthon and Barrett” assay (pH = 6.00), high activity was revealed at pH = 8.15. Lipoxygenases are therefore present in *S. marinoi*, though not active at pH = 6.00, but at seawater pH. Attempts to adapt the “Anhon and Barrett” method to FAH measurements at seawater pH were unsuccessful due to the pH requirement of the secondary reaction, precluding the use of this assay for species or strains whose LOX enzymes are not active at pH = 6.00. Another limitation of this assay was the instability of absorbance. Color formation continued after sodium dodecyl sulfate (SDS) was added to block LOX activity, which led to increasing absorbance values over time. It is, therefore, imperative to read absorbance at precise time intervals to obtain reproducible results with this assay, at least when working with diatom samples. This was likely one of the reasons for the high standard deviation observed among biological replicates. Another reason may lie in the metabolism of the diatoms themselves, as the results were more reproducible for *C. affinis* than for the other two species. Oxylipin production has been shown to vary with nutritional status [39,40] and growth phase [39,41] and slight metabolic differences in culture state may lead to large variations in metabolism.

Although LOX products have been shown to have a deleterious effect on the reproduction of copepods and other marine organisms [5,6], the relevance of PUAs and NVOs in shaping marine ecosystems is still under discussion. As LOXs are responsible for the first oxidation step, the study of its first reaction product can fundamentally contribute to understanding the function and role of these secondary metabolites in phytoplankton. This is especially true considering that FAHs are themselves deleterious for copepod development at concentrations comparable to those of PUAs [10]. Compared to other methods of FAH assessment, colorimetric assays are rapid, simple, and require only a spectrophotometer that is commonly used in many laboratories. They are therefore a valuable method for measuring the potential impact on copepod reproduction of cultured diatom samples and natural phytoplankton collected at sea during diatom blooms. The present study also shows that FAH measurements can be carried out on samples collected in the field if they are shock frozen in liquid nitrogen and kept at −80°C until analysis as this does not impair LOX activity.

The FOX2 colorimetric method proved to be superior to the “Anthon and Barrett” assay both in terms of sensitivity and replicability. By means of the FOX2 assay we found higher LOX activity in *C. affinis* and *S. marinoi* with respect to *T. rotula.* This is in accordance with our previous data on the reproductive success of copepods feeding on these diatom species [3]. Those studies, in fact, showed that *T. rotula* has a weaker negative effect than *S. marinoi* and *C. affinis* on the hatching success of copepod eggs when females feed on these species in the laboratory [4,10,42]. In particular, when the copepod *Temora stylifera* was fed on a diet of *S. marinoi* and *C. affinis*, naupliar production ceased altogether within only 3 days of feeding, while on a *T. rotula* diet egg viability never reached zero, although a decrease of 90% occurred after 14 days. A spectrophotometric assay cannot substitute the chemical analysis of LOX end-metabolites, but, on the other hand, the chemical analysis of PUAs and NVOs requires knowing all the relevant metabolites mediating the negative effect on copepods. Measuring only oxylipin production in certain diatom species may thus underestimate the effect of these microalgae on copepods, also considering that FAHs are themselves deleterious [10]. Indeed, oxylipin levels in *C. affinis* were similar to those of *T. rotula*, whereas *C. affinis* has a stronger negative effect on copepod reproduction, similar to *S. marinoi* [10]. This may also indicate that not all relevant molecules have been identified in *C. affinis.* The evaluation of FAH levels, which were similar between *S. marinoi* and *C. affinis*, gave a better evaluation of the toxic potential of these diatoms.

## 4. Experimental Section

### 4.1. Cell Culturing and Harvesting

For the present work, no specific permissions were required to collect samples of phytoplankton, the locations were not privately owned or protected in any way and no endangered or protected species were collected.

Batch cultures of *S. marinoi* (CCMP 2092), *C. affinis* (isolated in the Northern Adriatic Sea in 2002 by Francesco Esposito, Stazione Zoologica Anton Dohrn), and *T. rotula* (CCMP 1647) were grown in silicate-enriched Guillard’s f/2 medium prepared from 0.2 µm filtered and autoclaved seawater [43]. Cultures were grown in two-liter polycarbonate bottles and constantly bubbled with air filtered through 0.2 µm membrane filters (Sartorius, Goettingen, Germany). Cultures were kept in a climate chamber (RefCon, Napoli, Italy) at 20 °C on a 12 h:12 h light:dark cycle at 100 µmol photons m^−2^·s^−1^. Culture growth was monitored daily from samples fixed with one drop of Lugol and counted in a Bürker counting chamber under an Axioskop 2 microscope (20×) (Carl Zeiss GmbH, Jena, Germany). Cell cultures were harvested in stationary phase by centrifugation at 2200 *g* for 10 min at 4 °C using a cooled centrifuge with a swing-out rotor (DR 15P, Braun Biotechnology International, Allentown, PA, USA). As the assays should be applicable to samples collected at sea and stored, the effect of freezing the samples prior to analysis was examined. Half of the culture was centrifuged and analyzed immediately, whereas the other half was pelleted, frozen in liquid nitrogen, and kept at −80°C until analysis.

For analysis, the pellet was suspended in buffer solution (see sections 4.2 and 4.3 for details) and sonicated for 1 min with a micro tip at 20% output on ice (S-250A, Branson Ultrasonics, Danbury, CT, USA). Aliquots from the cell lysate were taken for LOX activity assays and protein determination, which was carried out according to the Bradford method (Bio-Rad, Hercules, CA, USA) with bovine serum albumin (BSA) as standard [44]. Statistical analyses were carried out with GraphPad Prism 4.00 (GraphPad Software, San Diego, CA, USA), *p* < 0.05 indicates a significant difference. To test for differences among more than two samples a one-way analysis of variance (ANOVA) was applied. When this delivered significant results, Tukey’s multiple comparison test was performed to compare every pair of means. All chemicals were purchased from Sigma-Aldrich (St. Louis, MO, USA) unless otherwise stated.

### 4.2. Colorimetric Assay According to the FOX2 Method

The FOX2 assay [23] was here optimized for diatom samples. The pellets were suspended in buffer solution at 10 × 10^6^ cells mL^−1^ for *S. marinoi* and 5 × 10^6^ cells mL^−1^ for *C. affinis* and *T. rotula* before sonication. The reagent mix was prepared by dissolving ferrous sulfate (2.5 mM final concentration) in perchloric acid (1.1 M final concentration). One volume of this concentrated reagent was added to nine volumes of HPLC grade methanol, containing 167 μM xylenol orange and 4.4 mM BHT to make a working reagent of 250 μM ferrous sulfate, 150 μM xylenol orange, 110 mM perchloric acid and 4 mM BHT in 90% *v*:*v* methanol.

Three aliquots of cell lysate corresponding to different cell amounts (from 7.5 × 10^4^ to 7.5 × 10^5^ cells) were added to appropriate volumes of buffer solution to obtain a final volume of 200 μL. Then, 800 μL of FOX2 reagent was added to each sample. Fifteen min after the addition of the reagent, the samples were centrifuged at 12,000 *g* for 4 min at 16 °C (Biofuge Fresco, Heraeus, Hanau, Germany) and the absorbance of the supernatants was measured at 560 nm in a spectrophotometer (8453, Hewlett Packard, Palo Alto, CA, USA) against a blank containing all the components of the FOX2 assay except the sample. Fatty acid hydroperoxide concentrations were calculated from a standard curve of commercial FAHs and normalized for protein.

The effect of pH and salt concentration on FAH production was assessed using *S. marinoi* as a model species. Deionized H_2_O (Milli-Q) and the buffers 50 mM Tris-HCl pH = 7.50, 50 mM Tris-HCl pH = 8.15 and 50 mM Tris-HCl 0.5 M NaCl pH = 8.15 were tested. In addition, to evaluate the kinetics of FAH formation, different reaction times were investigated. Immediately before sonication and after 5, 10 and 20 min from sonication, aliquots were withdrawn and analyzed with the FOX2 assay.

The specificity of the FOX2 assay for FAHs was improved using reducing agents. According to the procedure described by Nourooz–Zadeh and co-authors [37] absorbance at 560 nm due to FAHs can be differentiated from interfering aspecific absorbance by incubating a parallel series of samples with 40 μL 10 mM TPP in methanol. This reduces the FAHs to the corresponding hydroxyacids. The samples were stirred with TPP and incubated for 30 min to allow for the complete reduction of any –OOH present. Following the incubation, the assay was performed as usual. Samples without TPP were treated as described above except for the substitution of the TPP aliquot with methanol. Fatty acid hydroperoxide production was determined as the difference between TPP treated and untreated samples. Another reducing reagent, TCEP [38], soluble in aqueous media, was also tested. Following the procedure described above, samples were combined with 20 μL of 0.5 M TCEP, stirred and then incubated for 1 h before addition of the FOX2 reagent. Samples without TCEP were treated in the same way, except that the TCEP aliquot was substituted with buffer solution. All experiments were carried out in biological triplicate.

### 4.3. Colorimetric Assay According to Anthon and Barrett

This colorimetric assay is based on the detection of FAHs by the oxidative coupling of 3-methyl-2-benzothiazolinone (MBTH) with 3-(dimethylamino) benzoic acid (DMAB) in a reaction catalyzed by hemoglobin [21]. The pellets were suspended in deionized H_2_O (Milli-Q) at 1 mL·g^−1^ sample before sonication. The cell lysate (4 mg wet weight) was then incubated with 0.4 mL 10 mM DMAB and 0.1 mM ethylenediaminetetraacetic acid in 50 mM sodium phosphate buffer and 0.5 mL 0.2 mM MBTH containing 12.5 µg·mL^−1^ hemoglobin. The final pH of the reaction mixture was pH = 6.00. After 20 min SDS (0.3% final concentration) was added to stop the reaction. The samples were centrifuged at 10,000 *g* for 5 min at 4 °C in an Eppendorf ultracentrifuge and absorbance of the supernatant read at 598 nm in a spectrophotometer. Blanks were subtracted from absorbance readings. These consisted of the cell lysate added to the colorimetric reagents already containing 0.3% SDS, which were centrifuged and measured immediately. Fatty acid hydroperoxide concentrations were calculated from a standard curve of commercial FAHs. Six biological replicates were measured for *S. marinoi*, and four replicates for *C. affinis* and *T. rotula*.

### 4.4. Polarographic Assay

The oxygen consumption rate of the cell lysate was measured in a Gilson 5/6 oxygraph (Gilson Medical Electronics, Middleton, WI, USA) at 22 °C in a water-jacketed reaction vessel with a Clark electrode covered with a Teflon^®^ membrane [16]. Measurements were conducted in 0.2 M sodium borate buffer at pH = 8.15 or 0.2 M sodium phosphate buffer at pH = 6.00. Eicosapentaenoic acid (0.2 mM) was added to the cell lysate as external substrate, prepared according to Axelrod *et al.* [19] and LOX activity determined as the difference between oxygen consumption before and after EPA-addition.

### 4.5. Oxylipin Quantification

Thirty min after sonication of the pellet (suspended in Milli-Q), acetone (1:1 *v*:*v*) was added together with decenal and 16-hydroxy-hexadecanoic acid as internal standards. The H_2_O:acetone mixture was centrifuged at 2750 g at 4 °C for 6 min and the pellet extracted two more times with H_2_O:acetone at 1:1 *v*:*v*. The combined supernatants were extracted three times with CH_2_Cl_2_ (Carlo Erba, Milan, Italy) at 1:1 *v*:*v*. The organic phase was dried over Na_2_SO_4_, filtered, and the solvent was removed under reduced pressure at room temperature (Büchi Rotavapor R-114, Büchi Laboratory Equipment, Flawil, Switzerland). Extracts were in part derivatised with (1-ethoxycarbonylethyliden)-triphenyl-phosphorane at 1.1 mg:1 mg extract in CH_2_Cl_2_ for 20 h at room temperature according to d’Ippolito *et al.* [42] for analysis of volatile PUAs on a GC-MS (Focus GC-PolarisQ, Thermo Scientific, Waltham, MA, USA). According to reference [20], the remaining extract was derivatized with ethereal diazomethane for analysis of NVOs on a Qtof-*micro* mass spectrometer (Waters SpA, Milan, Italy), equipped with an electrospray ionization (ESI) source (positive mode) and coupled to a HPLC system (Waters Alliance, Milford, MA, USA). Seven biological replicates were processed for *S. marinoi* and four for *C. affinis* and *T. rotula*. The oxylipins (PUAs, NVOs) quantified in the present study, were already identified previously [12,20,45].

## 5. Conclusions

Using the FOX2 assay to assess FAH production in diatom lysates gave good results that are corroborated by the available literature data on species-specific negative effects on copepod reproductive success. We, thus, propose the FOX2 assay as a valid method for measuring the potential toxic impact of cultured diatoms and natural phytoplankton on copepod reproduction and as a useful tool that may contribute to understanding copepod–diatom interactions at sea.

## Supplementary Files

Supplementary File 1
