# Supplementary material for: Determination of Lipid Hydroperoxides in Marine Diatoms by the FOX2 Assay
Source: Mar Drugs. 2015 Sep 11;13(9):5767–83. doi: 10.3390/md13095767 (PMC4584353; doi:10.3390/md13095767)
Supplement: Supplementary File 1 [file marinedrugs-13-05767-s001.docx]

Supplementary Information

**Figure S1.** Time course of hydroperoxide production in *Skeletonema marinoi* lysate measured by the FOX2 assay (shown as absorbance at 560 nm). One aliquot was taken immediately prior to sonication (*t* = 0 min) and the others at 5, 10 and 20 min after sonication. Data represent mean ± SD of three biological replicates.

**Figure S2.** Spectrophotometric scan from 300 to 700 nm. The FOX2 reagent before (dashed line) and after (continuous line) the addition of *Skeletonema marinoi* lysate. The dotted plot refers to diluted *S. marinoi* lysate in 50 mM Tris-HCl 0.5 M NaCl pH = 8.15.


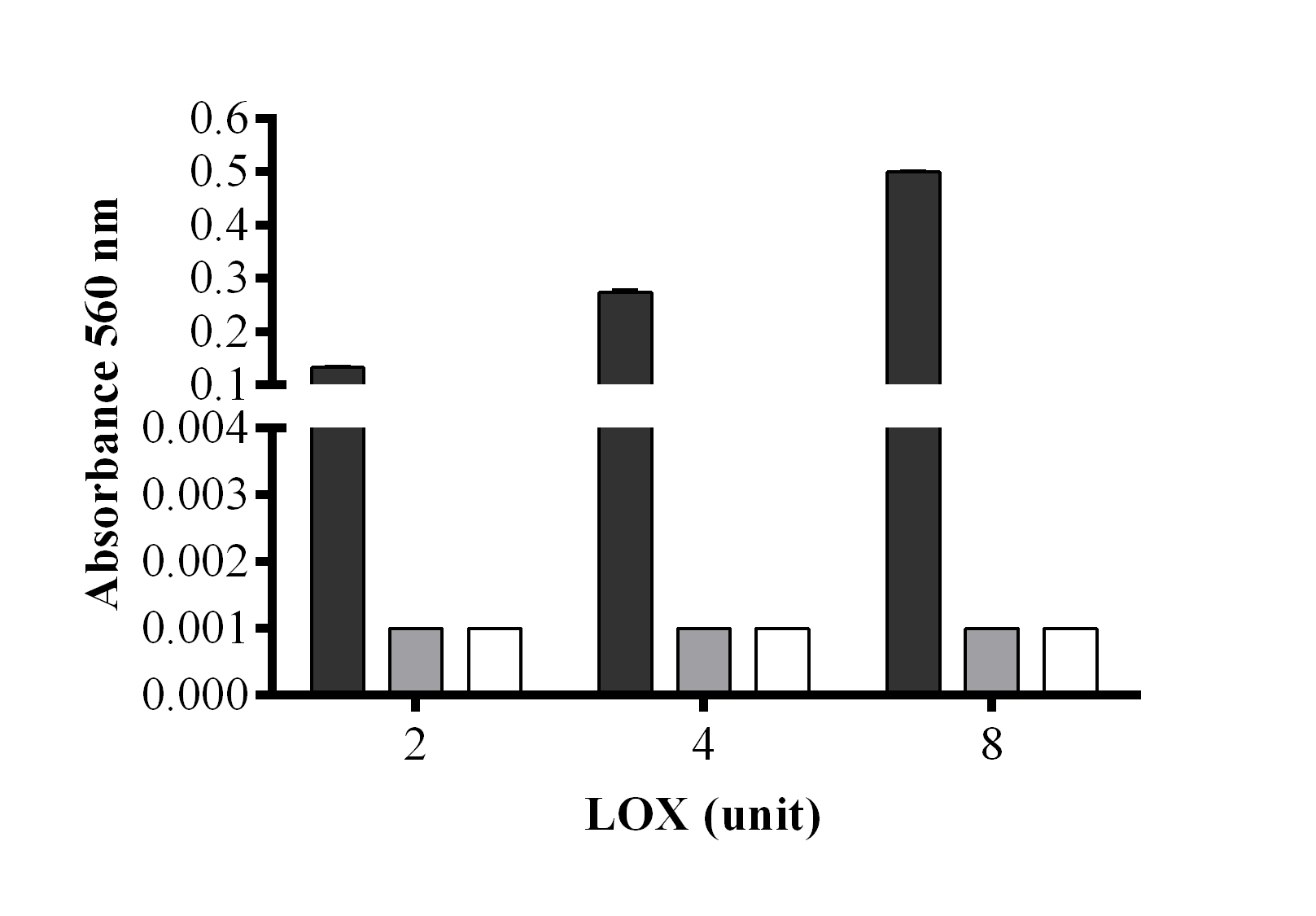


**Figure S3.** Effect of reducing reagents on hydroperoxide production by pure lipoxygenase (LOX) enzyme. Aliquots of pure LOX enzyme were incubated without (black), and with the reducing reagent triphenylphosphine (TPP, gray) or tris(2-carboxyethyl)phosphine (TCEP, white) at different concentrations for 10 min with 16 mM linoleic acid as substrate. Data represent mean + SD of three biological replicates.


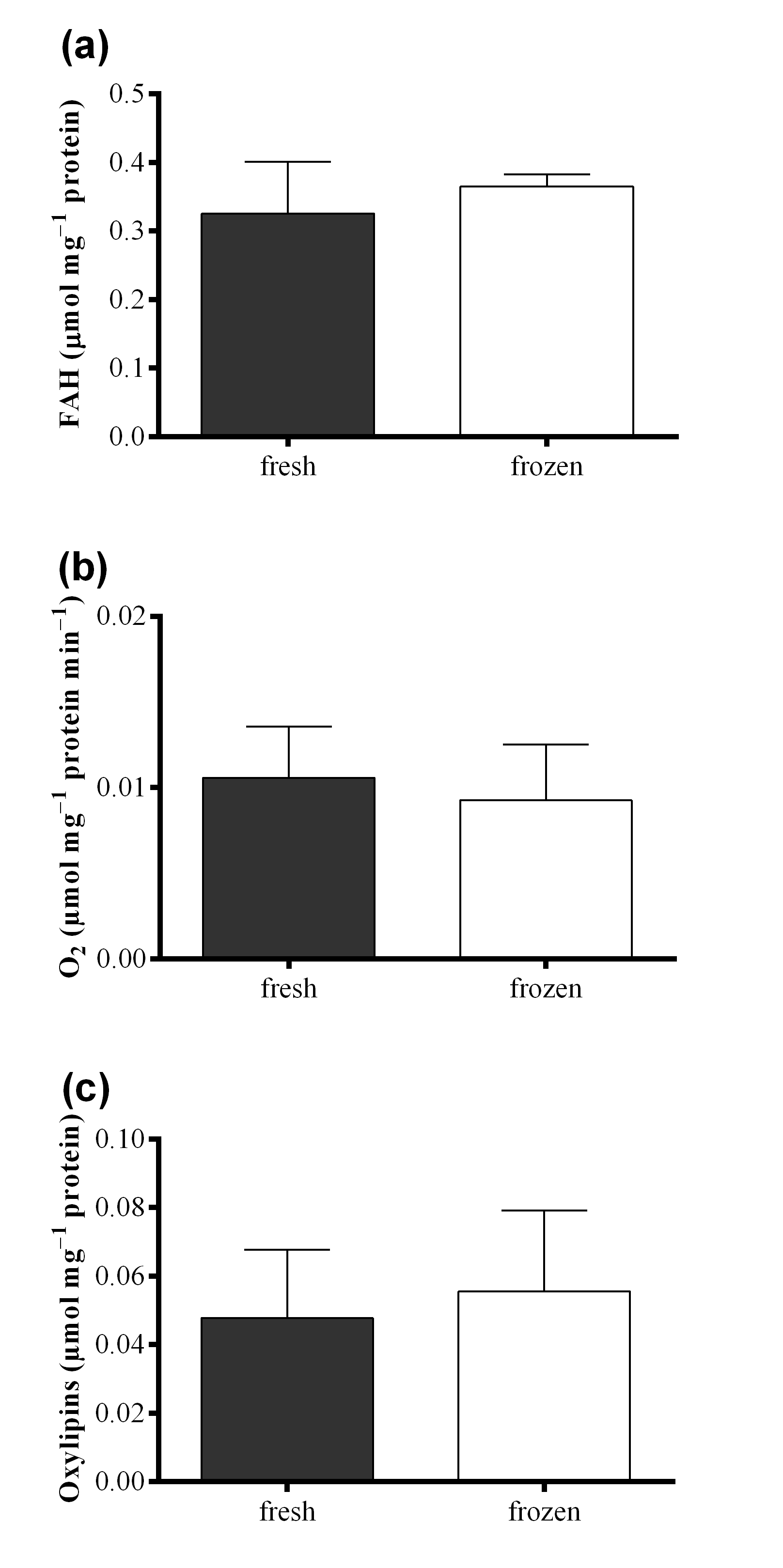


**Figure S4.** Comparison between fresh (black) and frozen (white) diatom samples. Fatty acid hydroperoxide production measured in samples of *Chaetoceros affinis* by (**a**) the “Anthon and Barrett” assay, (**b**) polarographic measurement and (**c**) oxylipin levels. Data represent the mean + SD of four biological replicates.
